# Effects of Photodynamic Therapy with Redaporfin on Tumor Oxygenation and Blood Flow in a Lung Cancer Mouse Model

**DOI:** 10.1038/s41598-019-49064-6

**Published:** 2019-09-02

**Authors:** Malwina Karwicka, Barbara Pucelik, Michał Gonet, Martyna Elas, Janusz M. Dąbrowski

**Affiliations:** 1 Jagiellonian University, Faculty of Biochemistry, Biophysics and Biotechnology, Gronostajowa 7, 30-387 Kraków, Poland; 2 Jagiellonian University, Faculty of Chemistry, Gronostajowa 2, 30-387 Kraków, Poland; 3 Jagiellonian University, Małopolska Centre of Biotechnology, Gronostajowa 7A, 30-387 Kraków, Poland

**Keywords:** Lung cancer, Photobiology

## Abstract

Three photodynamic therapy (PDT) protocols with 15 min, 3 h and 72 h drug-to-light time intervals (DLIs) were performed using a bacteriochlorin named redaporfin, as a photosensitizer. Blood flow and pO_2_ changes after applying these protocols were investigated in a Lewis lung carcinoma (LLC) mouse model and correlated with long-term tumor responses. In addition, cellular uptake, cytotoxicity and photocytotoxicity of redaporfin in LLC cells were evaluated. Our *in vitro* tests revealed negligible cytotoxicity, significant cellular uptake, generation of singlet oxygen, superoxide ion and hydroxyl radicals in the cells and changes in the mechanism of cell death as a function of the light dose. Results of *in vivo* studies showed that treatment focused on vascular destruction (V-PDT) leads to a highly effective long-term antineoplastic response mediated by a strong deprivation of blood supply. Tumors in 67% of the LLC bearing mice treated with V-PDT regressed completely and did not reappear for over 1 year. This significant efficacy can be attributed to photosensitizer (PS) properties as well as distribution and accurate control of oxygen level and density of vessels before and after PDT. V-PDT has a greater potential for success than treatment based on longer DLIs as usually applied in clinical practice.

## Introduction

Lung cancer is one of the most common types of malignant tumor worldwide and the leading cause of cancer-related deaths. Despite many improvements in disease diagnosis and management, chemotherapy is still the mainstay of treatment for both non-small cell lung cancer (NSCLC) and small cell lung cancer (SCLC). However, chemotherapy resistance can limit the ability to effectively treat advanced lung cancer^1,2^. New anticancer strategies are urgently needed to improve therapeutic success of lung cancer treatments beyond conventional chemotherapeutic drug outcomes.

Photodynamic therapy (PDT) is recognized in clinical practice and holds considerable promise for many solid tumors, including lung cancer^3^. PDT employs reactive oxygen species (ROS) generation according to two main photochemical processes (type I: photo-induced electron transfer with generation of oxygen-centered radicals and type II: energy transfer with singlet oxygen production)^4–6^. ROS are involved in the oxidation of biological structures, leading to oxidative stress and consequently to: (i) apoptosis, necrosis and/or autophagy, (ii) closure of tumor blood vessels^7^, (iii) stimulation of the immune system to local and systemic immune responses^8,9^. The contribution of each mechanisms and the final therapeutic efficacy depend on the dose and structure of the photosensitizer (PS), the therapeutic protocols used and the time intervals from the administration of PS to irradiation (drug-to-light intervals; DLIs), radiation power and oxygen concentration in the tumor^4,10–13^. Results from several *in vivo* studies performed using lung cancer models indicate variable tumor responses to PDT. Korbelik and colleagues showed that activation of the complement system by Photofrin-PDT in a Lewis lung carcinoma (LLC) tumor model appears to reflect the natural response of innate immunity engaging to maintain homeostasis following acute tumor injury^14^. The antitumor effect of PDT against LLC in *in vivo* models can be potentiated by 5-aza-dC^15^. Moreover, PDT with 5-aminolevulinic acid (ALA-PDT) decreased metastasis of cancer cells *in vivo*^16^. Application of ALA-PDT lowered the rate of metastatic spreading, decreased vascular endothelial growth factor (VEGF) levels in blood serum of LLC bearing mice, and resulted in morphologic alterations of vascular system in tumor tissue. Recently, Allison and colleagues described the mechanisms of PDT action with different photosensitizers (PS) and light sources in the treatment of many tumors including lung cancer^17^. Loewen *et al*. reviewed the use of Photofrin and HPPH (2-[1-hexyloxyethyl]-2 devinyl pyropheophorbide) in treatment of early-stage lung cancer and reported clinical results. PDT with HPPH led to the delayed cellular effects within tumor and thus was indicated for the palliation of endobronchial lung cancers^18^.

Many treatment failures observed after therapy may be attributed to intrinsic tumor resistance. Tumor hypoxia may be a potential contributing factor to treatment resistance and its impact on tumor vasculature - also highlighted as an important target for PDT^19^. Recently, the importance of monitoring and non-invasive visualization of tumor blood microvasculature in real time was indicated in photodynamic treatment mediated by chlorine e6 and Photoditazin-based photosensitizer^20^. It has been reported that PDT induces hypoxia and expression of VEGF^21^ *via* the HIF-1α pathway^22–24^. Hypoxia is found in 50–60% of locally advanced solid tumors and is associated with poor clinical outcomes^25,26^. Normal physiological oxygen partial pressure (pO_2_) varies among different tissues, but is usually measured at approximately 43 mmHg. Tumor pO_2_ is a critical factor related to negative effects on cellular functionality depending on corresponding normal tissue pO_2_ and is proposed to range between approximately 8–10 mmHg in most tumors. However, the critical pO_2_ threshold associated with treatment resistance may vary with therapeutic schemes^25^. Although a satisfactory effect in treating superficial tumors may be obtained, PDT efficacy in a hypoxic environment is still impaired. PDT increases hypoxia via oxygen consumption and vascular shutdown effects. Low oxygen content in a tumor can reduce phototoxicity, preventing PDT from achieving its full therapeutic potential^27^. The efficacy of ^1^O_2_ generation is fundamentally dependent on O_2_ concentration, thus low O_2_ levels would hamper PDT treatment with a PS that generates mainly ^1^O_2_. Complete death of radiation-induced fibrosarcoma (RIF) cells after a photodynamic effect mediated by Photofrin appears to be achieved at levels of normal tissue oxygenation with no increase in effectiveness at higher O_2_ concentrations^28^. Furthermore, lowering O_2_ levels below 5% appears to progressively limit cellular photodamage with a half-value of about 1% (i.e. appr. 7 mmHg). Traditional methods have attempted to optimize tumor oxygenation to maximize PDT efficacy.

Current understanding of the techniques that can effectively reverse tumor oxygen content during PDT is limited. Therefore, optimizing treatment efficacy under limited oxygen conditions is of great importance for PDT. Tong, X. *et al*. performed dynamic PET using hypoxic specific tracer ^18^F-FMISO combined with pharmacokinetics modeling. Their research provided the information of hypoxia status during PDT in two tumor xenograft models (U87MG and MDA-MB-435) and indicate that this approach offers a potential imaging tool to characterize tumor hypoxia during treatment^29^. Y. Cheng *et al*. reported a novel method of “oxygen self-enriching PDT” to enhance the ^1^O_2_ generation of an applied PS^30^. Although the tumor oxygen content remained limited during PDT, sufficient O_2_ was made available for photodynamic consumption by the loaded PS, resulting in improved efficacy. This type of enhancement is possible regardless of pre-existing hypoxia, photodynamic consumption, or vascular damage. Moreover, ^1^O_2_ lifetime in perfluorocarbon nanodroplets (PFC) is longer than in a cellular environment or in water, which results in long-lasting photodynamic effects^27^. Tumor tissue oxygenation levels are even more crucial after PDT treatment as they influence overall therapeutic outcome. We have shown that redaporfin-PDT induced mild and transient hypoxia with a drug-to-light time interval (DLI) of 72 h and led to intense pO_2_ compensatory effects and modest tumor inhibition in a S91 melanoma model^31^. In contrast, strong and persistent local hypoxia after vascular-targeted PDT (V-PDT; DLI = 15 min) caused tumor growth inhibition and led to long-term survival. Non-invasive molecular imaging approaches targeting hypoxia are still being developed and have shown some early success in preclinical and clinical settings^32–35^. These methods also provide staging information and can be useful for monitoring behavior and tumor response *in vivo*^36–38^.

Many of innovative nanoparticle-based photosensitizers developed recently (e.g. nanoformulation by coating nanoclusters of SPIONs with the photosensitizer Ce6) are also able to serve as effective dual-mode agents for theranostics and photodynamic therapy of murine tumor models^39–42^. Small animal Electron Paramagnetic Resonance (EPR) imaging modalities have been applied e.g. in time-domain mode and EPR imaging is used routinely in mouse models of tumor to map tumor hypoxia^43^. As mentioned above, the importance of mapping out tumor hypoxia stems from the fact that hypoxic tumor cells are about 3–4 times more resistant to various types of treatments than normal cells. Thus, mapping of tumor hypoxia aids in the planning of therapeutic procedures and improves prognosis of treatment^44,45^. To address this challenge, our study employed three-dimensional imaging using EPR oximetry to assess tumor response to PDT with redaporfin as a PS. Redaporfin is currently in phase II clinical trials for advanced head and neck tumors. The phototoxicity of this PS is mediated by both type I and type II photochemical mechanisms, so it can efficiently generate singlet oxygen as well as oxygen-centered radicals after irradiation with near-infrared radiation (NIR) light. Previously, we demonstrated the superior efficacy of redaporfin as a PS in multiple tumor models^4,46,47^, including highly aggressive pigmented melanoma^48^.

Herein, we performed *in vivo* PDT on LLC tumors growing in syngeneic C57BL/6J mice. LLC was selected for the tumor model as it is considered highly malignant, life-threatening, and one of the most difficult cancers to treat^49^. An advantage of the LLC model is that implanted cells are immunologically compatible with the murine system, unlike widely used xenograft models in which human cells are implanted into mouse tissue. The LLC model can be created on an immunocompetent murine background, such as C57BL, and true immune and toxicity responses can be evaluated with respect to targeted therapies and tumor growth^50,51^. Current study found that a single dose of redaporfin and irradiation with NIR light at a dose of 105 J/cm^2^ can effectively inhibit LLC tumor growth. It has been demonstrated that redaporfin can accumulate in tumor tissue at 15 min after intravenous (*i.v*.) injection of PS. Additionally, we assessed intracellular hypoxia in tumor tissue by a non-invasive EPR oximetry technique using a paramagnetic probe. This approach uniquely allows for measuring changes in tumor tissue pO_2_ and reperfusion in LLC tumor models. Moreover, we have studied the level of oxygen in LLC tumors using pimonidazole hydrochloride as an immunohistochemical marker for hypoxia and correlated tissue oxygenation, tumor vasculature, and blood flow with overall therapeutic efficacy after redaporfin-PDT.

## Results

### The *in vitro* studies of redaporfin activity against LLC cells

**Figure 1:**
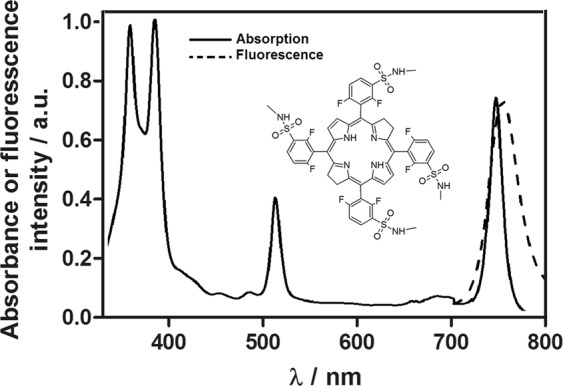
Electronic absorption and fluorescence spectra of redaporfin and its molecular structure (inset).

Redaporfin readily absorbs NIR light (Fig. 1) in the middle of the phototherapeutic window where endogenous pigments do not absorb light, photons are non-toxic and their energy is still sufficient for photochemical reactions such as an energy transfer to molecular oxygen.

**Figure 2:**
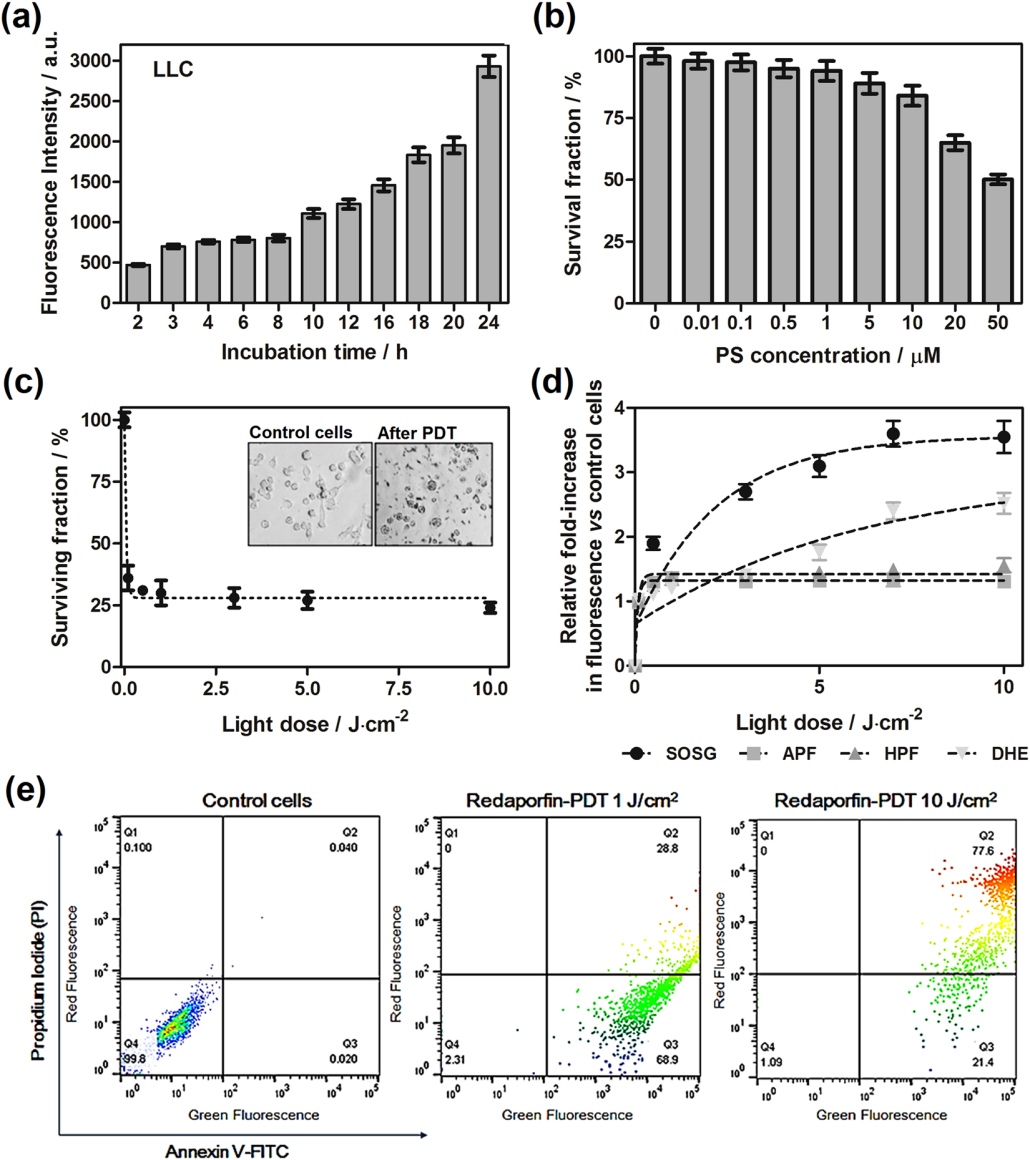
*In vitro* evaluation of redaporfin efficacy in LLC cells: (**a**) time-dependent cellular uptake of redaporfin in LLC cells; (**b**) cytotoxicity in the dark induced by redaporfin in a wide-range of applied concentrations; (**c**) redaporfin-mediated photodynamic effect against LLC cells performed after 3 h incubation and irradiation with various light doses, inset shows morphology of cells before and after treatment; (**d**) photogeneration of ROS in LLC cells: fluorescence generated from ROS probes: APF, HPF, SOSG and DHE in LLC cells incubated with redaporfin for 3 h and irradiated with NIR light; (**e**) determination of cell death mechanism induced by photodynamic effect: the Annexin V-FITC/propidium iodide (PI) double staining assay (Annexin V-FITC in green fluorescence and PI in red fluorescence) was used to detect phosphatidylserine externalization in apoptosis and analyze the membrane integrity: non-treated control LLC cells (left), LLC cells treated with redaporfin-PDT with light dose at 1 J/cm^2^ (middle) and 10 J/cm^2^ (right).

In order to determine the photodynamic efficacy of F_2_BMet, in the first part of the study, the biological activity *in vitro* was analyzed. Firstly, we investigated the time-dependent cellular uptake of redaporfin by LLC cells exposing the cells to redaporfin at concentration of 5 µM. As shown in Fig. 2a, the cellular uptake attains its maximum after 24 h of incubation and it is comparable to that performed on other cell lines^31^. The photoxicity in the dark towards LLC cells was tested with various incubation times. Incubation of the cells for 24 h with redaporfin in the range of concentrations tested (0.1–100 µM) did not reveal significant cytotoxicity up to dose of 20 µM (Fig. 2b).

It is well-known that cell death triggered by photodynamic therapy occurs through various mechanisms, including apoptosis, necrosis, autophagy and others^52^. Upon irradiation of LLC cells with the light dose of 0.15 J/cm^2^ (λ = 735 ± 20 nm), redaporfin generates ROS that consequently lead to cell destruction and significant cell killing (>80%) (Fig. 2c). Cell death mechanism was further examined by flow cytometry using Annexin V-FITC and PI staining, Fig. 2e. It was observed that redaporfin-mediated photodynamic effect against LLC cells with high light dose (10 J/cm^2^) caused necrosis, while smaller doses (1 J/cm^2^) initiated apoptosis. It was revealed that irradiation of redaporfin-photosensitized cells led to an increase in the number of cells that expressed phosphatidilserine from the inner space of the plasma membrane to the cell surface combined with Annexin V-FITC (68.9% stained (Q3) vs. 2.31% non-stained, lived cells (Q4)). Furthermore, increasing the light dose led to a significant increase in Annexin V-PI double-positive and PI-positive cells, which suggested a decrease in the integrity of the cell membrane and switch to preferred necrotic cell death (77.6% stained cells (Q2) *vs*. 1.09% non-stained cells (Q4)). Redaporfin belongs to the group of compounds generating both singlet oxygen and oxygen-centered radicals. Nevertheless, the participation of photosensitizing mechanisms is dependent on the oxygen concentration in the reaction environment. The intracellular ROS generation by redaporfin in LLC cells was examined using SOSG probe selective for singlet oxygen production, APF and HPF specific for hydroxyl radicals and DHE for superoxide anion identification. All of applied probes are non-fluorescent, but their oxidized by ROS products emit a specific fluorescence. The non-irradiated cells did not show any fluorescein emission. As illustrated in Fig. 2d, during irradiation LLC cells, the singlet oxygen is produced in large amount what may indicate that the II type of reaction is more favorable mechanism of ROS generation in well oxygenated cellular environment. However, radicals such as the O_2_**˙**^**−**^ and HO**˙**, are most likely involved in the cell damage. Type I reactions triggered by redaporfin add to the Type II reaction initiated by singlet oxygen, which will have implications in *in vivo* conditions, where other factors such as hypoxia, blood flow, level of vascularization have to be taken into account.

### *In vivo* tissue distribution of redaporfin is dependent on DLI – a case study

**Figure 3:**
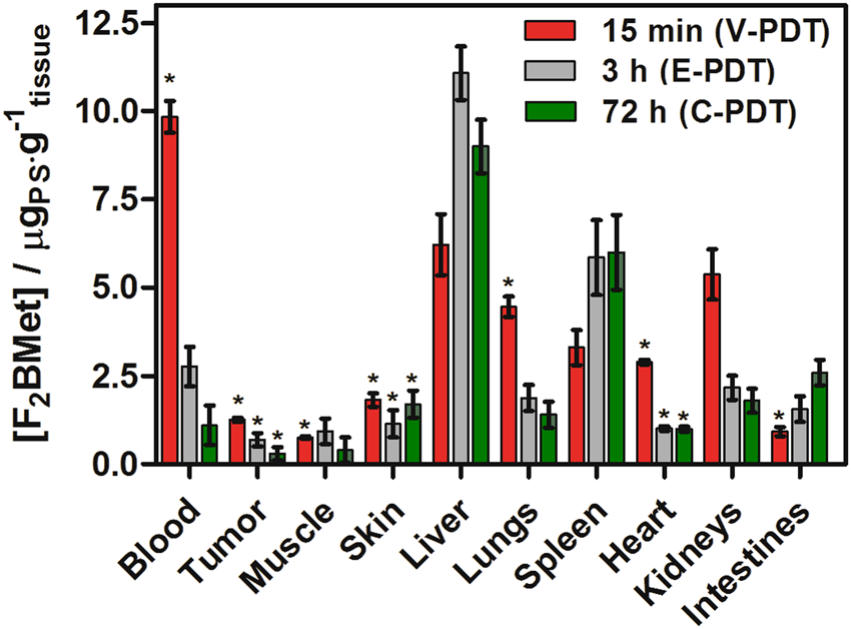
Biodistribution of redaporfin in blood, tumor and other tissues at: 15 min (red), 3 h post-injection (gray) and 72 h post-injection (green). Average values are shown with error bars representing the SEM. Results were considered as statistically significant with a confidence level of 95% (p < 0.05).

The experiments described in this section are focused on LLC tumor response and vascular effects after redaporfin-PDT. PDT can be designed to target either tumor vasculature, tumor cells, or both depending on PS properties^53^, formulation, and the DLI^54–56^ properly designed in order to increase therapeutic response. Tissue distribution of redaporfin in tumor, blood, and other organs were examined by fluorescence measurements of tissue extracts collected at 15 min, 3 h and 72 h after PS administration and results are presented in Fig. 3.

During the first 15 min post-injection, a higher amount of PS is found in blood and tumor than after 3 or 72 h. PS concentration increases in spleen, liver and intestines over time. Likewise after 72 hours the highest level of PS was identified in these organs, namely in the liver, spleen and intestines, respectively. This suggests that the main route of its elimination seems to be intestines. The biodistribution results are in good agreement with our previous studies performed on CT26^4,46^. A similar tissue distribution of redaporfin formulated with CrEL, was recently found in the same mouse model (C57BL/6J) bearing pigmented B16 melanoma^48^ as well as S91-bearing DBA/2 mice^31,57,58^ although the comparison must take into account varied vascularization of these models and different mode of administration employed. The biodistribution of redaporfin in C57BL/6J mice bearing LLC tumors also revealed that tumor-to-muscle (T:M) and tumor-to-skin (T:S) ratios achieved reasonable values already 15 min after *i.v*. injection of PS (Table 1).

**Table 1:** Tumor-to-Muscle (T/M) and Tumor-to-Skin (T/S) ratios of redaporfin at various DLIs obtained from C57BL/6J mice bearing LLC tumors.

	DLI = 15 min	DLI = 3 h	DLI = 72 h
T:M	1.68	0.74	0.75
T:S	0.69	0.60	0.18

**Figure 4:**
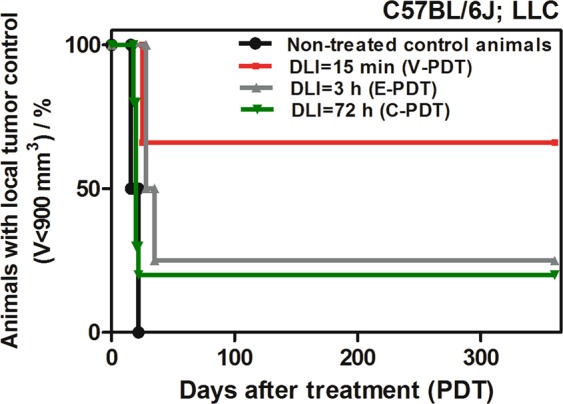
Kaplan–Meier analysis of the untreated mice and mice treated with redaporfin-PDT with time interval: 15 min (N = 6), 3 h and 72 h (N = 7).

In accordance with biodistribution studies, PDT efficacy with redaporfin was assessed in the same animal and tumor models, C57BL/6J mice bearing LLC, which were treated with a single dose of light at 15 min (V-PDT), 3 h (E-PDT) and 72 h (C-PDT) after *i.v*. injection of PS. Irradiation was carried out for 13.5 min, at a light dose of 105 J/cm^2^ using a 130 mW laser at 749 nm within the optimal safety margin for normal tissue. In order to evaluate tumor response related to the selected PDT protocols, the kinetics of tumor growth were investigated. Various acute reactions including edema, erythema, and scab formation were also noted after PDT. Local tumor responses after V-PDT and E-PDT differed. Edema appeared immediately after V-PDT treatment in contrast to a less acute inflammatory response following E-PDT and mild inflammatory reaction after C-PDT. Therapeutic success was estimated using Kaplan-Meier analysis, which presents the survival data of mice within a specified amount of time after PDT (Fig. 4).

In all PDT-treated mice, tumor growth was delayed compared to the control group and, consequently, partial or total tumor remission occurred. Tumor regrowth was observed up to 2 months after therapy especially after C-PDT, in half of the mice after E-PDT and in approximately 33% of the mice after V-PDT. Importantly, after this period of time a completed permanent tumor remission was achieved. Furthermore, therapeutic efficacy after V-PDT was much better (approximately 65% cure rate) than following E-PDT or C-PDT, for which only about 25% long-term cure rate was observed. Although cellular-targeted treatment like C-PDT or E-PDT failed to induce as effective tumor regression as V-PDT, it did retard tumor growth. This effect may be related to its relatively low PS concentration at 3 h or 72 h post-injection, as well as a less favorable biodistribution that may be insufficient to achieve PDT efficacy. V-PDT is therefore more effective therapeutic approach using redaporfin as photosensitizer than E-PDT or C-PDT. In E-PDT regimen light is delivered within 15 min after the photosensitizer intravenous administration, while it is still present within the vascular compartment. These results are also in an agreement with biodistribution studies indicating that tumor-to-muscle or to skin tissue ratios reach the highest values after 15 min than 3 h or 72 h post-*i.v*. injection of the photosensitizer. Nevertheless, the results of *in vivo* experiments indicate that the use of PDT against LLC tumors increases the average lifetime of animals in all three experimental groups. In the case of control tumors, this time is only 15 days. Subjecting tumor to F_2_BMet-PDT leads to about 20% of long-term cures in the case of a protocol with DLI = 72 h, 25% for DLI = 3 h and 67% in the case of DLI = 15 min. Significant differences can be seen in the initial observation period - LLC tumors treated with E-PDT are characterized initially by a good response to the therapeutic protocol used (swelling and edema), and tumor recurrences are observed approximately 30 days after the PDT. Such low PDT efficacy in C-PDT or E-PDT is surprising, due to delivered light doses, which in the applied schemes were significantly higher (>100 J/cm^2^) in comparison to the doses normally used for F_2_BMet-PDT (74 J/cm^2^)^46–48^.

Differences in the effectiveness of therapy result primarily from the selected tumor model, which is characterized by a very high degree of invasiveness, fast growth and relatively low vascularity. It is a model classified as one of the most resistant to treatment, which is also confirmed by the results obtained in the present work - despite protocols targeting cancer cells, which theoretically should prove effective, the percentage of cured animals was only 20%. This effect may result from poor tumor tissue perfusion, resulting in weak PS penetration of the tumor.

### Vascular Perfusion and hypoxic areas in LLC tumors after PDT

**Figure 5:**
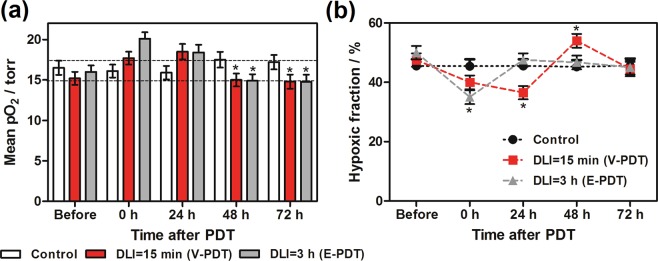
Changes in the oxygenation of tumors, determined from oxygen maps in control animals (**a**) N: 4–7 in each time interval and PDT-treated groups: with DLI = 15 min, N: 4–9 and DLI = 3 h, N: 4–10. (**b**) Hypoxic area was calculated as percentage of pixels with pO_2_ value less or equal to 10 torr in each tumor pO_2_ map. Results were considered as statistically significant with a confidence level of 95% (p < 0.05).

PDT causes rapid vascular damage, providing that a short drug to light period is applied. This effect was expected to produce a dramatic decrease in tumor tissue oxygenation. Surprisingly, oximetric maps of tumors (Fig. 5) revealed an increase in pO_2_ values and a decrease in HF10 immediately after treatment. Mean pO_2_ increased from approximately 15 torr to approximately 20 torr immediately after treatment, then at 48 h returned to the initial level.

**Figure 6:**
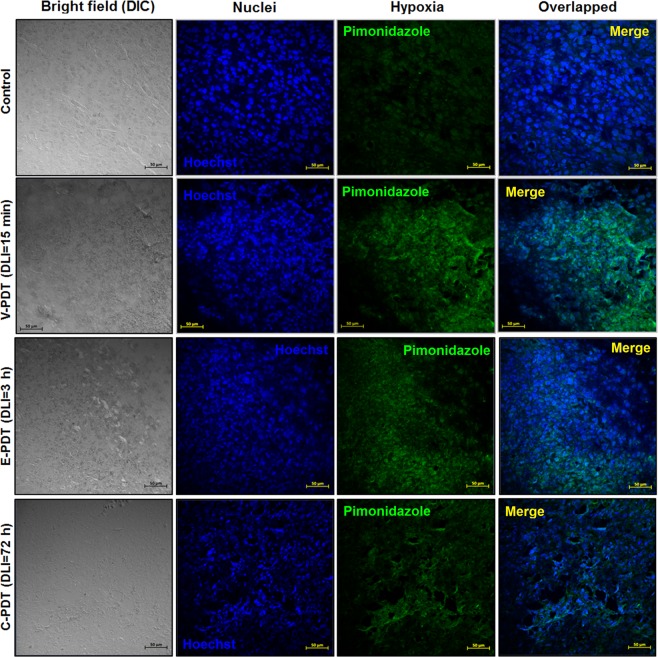
Pimonidazole staining of LLC tumor tissue, before (control) and 2 h after PDT. Hypoxic region stained by pimonidazole is shown in green fluorescence signal.

**Figure 7:**
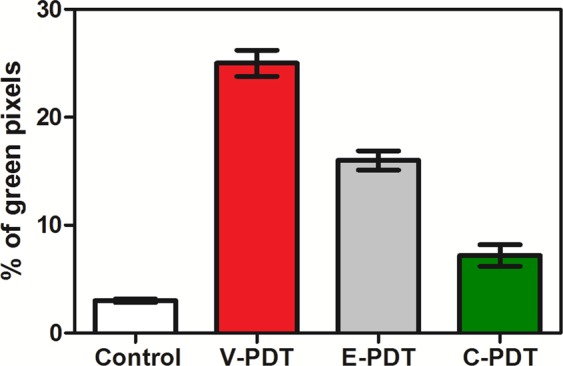
Changes in hypoxic area (pimonidazole positive) 2 h after PDT determined from IHC imaging. Values present the ratio of the sum of green pixels to the total tumor area in each image, with SEM.

Similarly, the hypoxic fraction determined from EPR imaging in untreated tumors was approximately 50%, after PDT it decreased to 35%, and then returned to initial levels. Furthermore, to assess PDT-induced hypoxic area in LLC tumors, the Hypoxyprobe™ (pimonidazole hydrochloride) was used and immunohistochemical analysis was performed. Pimonidazole is a 2-nitroimidazole that is reductively activated specifically in hypoxic cells and forms stable adducts with thiol groups in proteins, peptides, and amino acids^29^. It is worthy to noticing that amount of pimonidazole that is detected in tissue is directly proportional to the level of hypoxia within tumors. Figure 6 shows relatively low hypoxic area in LLC untreated tumors. However, after PDT treatment the hypoxia were more prominent especially for V-PDT. The increase in hypoxic area after V-PDT attains a higher value than after E-PDT or C-PDT. At 2 h post PDT, the hypoxic fraction increased significantly and reached the highest values for V-PDT, followed by E-PDT (moderate) and C-PDT that is notable as intense green spots (see Fig. 7). Moreover, after PDT tumor hypoxia was more heterogeneous in comparison with relatively more homogeneous enhancement distribution pattern observed for untreated control tumors.

Based on the image analysis we have also estimated the fraction of positively staining section (percentage of green pixels determined from the ratio of the sum of green pixels to the total tumor area in each image), Fig. 7. The mice treated with V-PDT and E-PDT showed a significant increase in pimonidazole accumulation (26.2% and 16.1%, respectively) 2 h after PDT. These results indicate the increase in the PDT-induced hypoxia immediately after the treatment.

**Figure 8:**
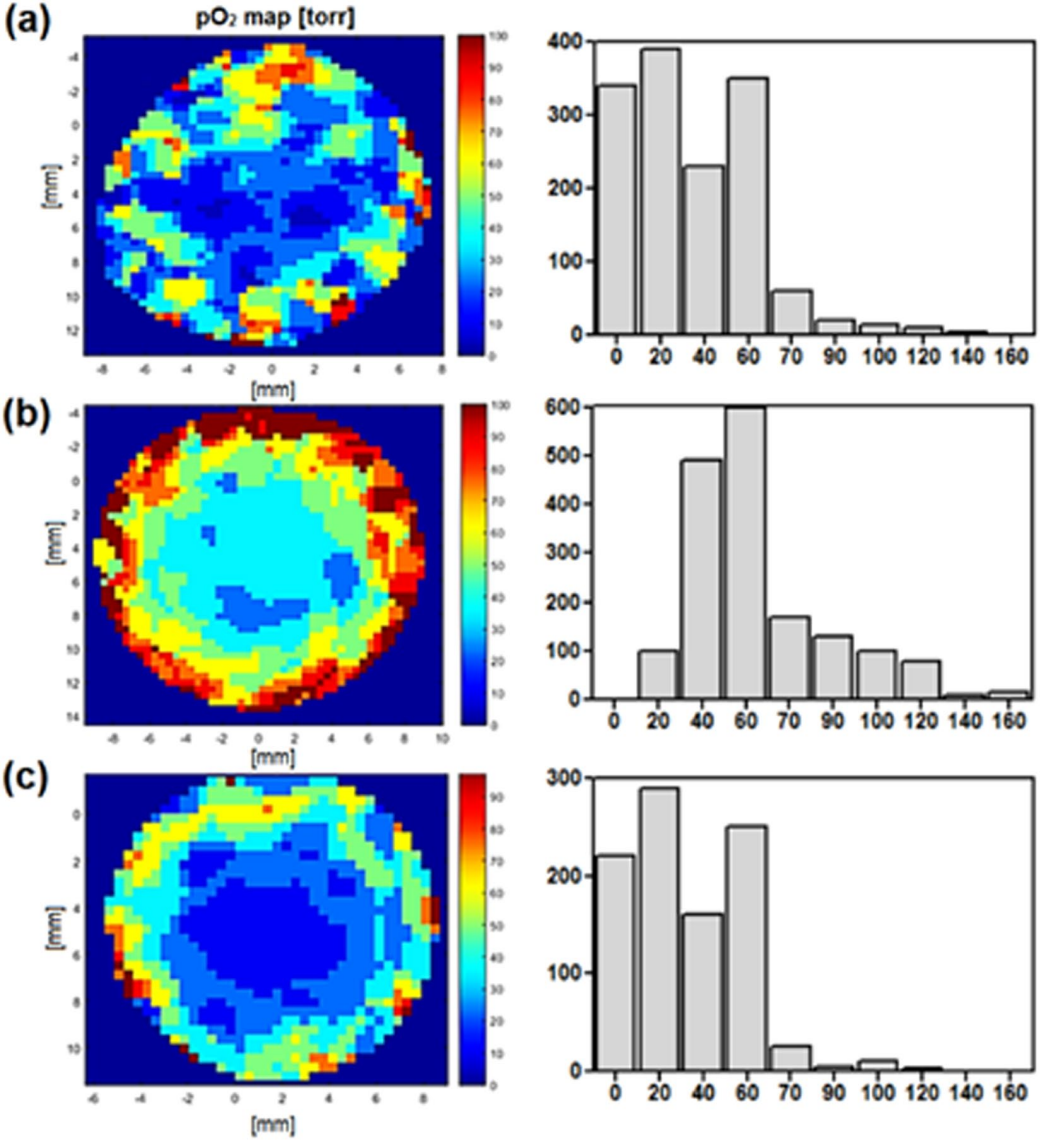
Representative oxygen maps of the area of interest (tumor is in the central part) with corresponding histogram presentation of pO_2_ level: A - before, B – 24 h and C – 72 h after illumination. Color bars show the pO_2_ scale from 0 (dark blue) to 100 mm Hg (red). Histograms show the distribution of the pO_2_ values from the individual pixels, with a distinct shift towards higher oxygenation at 24 h after V-PDT.

Furthermore, to better understanding the short-term and acute effects observed after photodynamic treatment, in the next stage of studies, we examined the tumor oxygenation and vascular effect after V-PDT and E-PDT. The detailed examination were performed especially for V-PDT - the phototherapeutic protocol characterized by the highest therapeutic efficacy (see Fig. 4.) The results were corroborated by more in-depth analysis of two-dimensional oxygen maps. The maps were acquired from the tumor area using a surface coil. Depth sensitivity was up to 6 mm and each pixel in the map represents pO_2_ averaged over this depth. As tumor size was approximately 3–4 mm, the maps encompassed the whole tumor volume. The mean pO_2_ values (Fig. 8) are averaged across the whole map, whereas the histograms (Fig. 8) represent the distribution of pO_2_ values from individual pixels.

**Figure 9:**
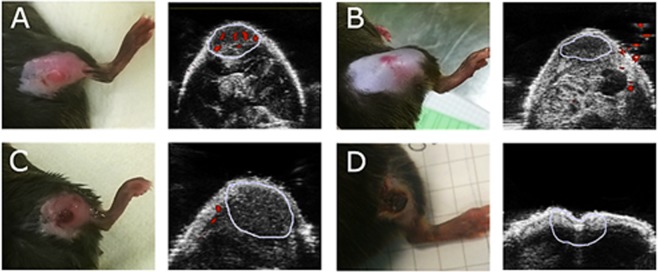
Representative photographs (left) and Power Doppler images (right) of tumors growing in the mouse leg. A-before, B- immediately after, C-24 h after and D-48 h after treatment (V-PDT). The area marked in blue corresponds to tumor or region of interest at each time point. Red shows the blood flow.

The histograms show a significant shift towards increased pO_2_ values in of treated tumor at 24 h, which again shift back towards lower values at 48 and 72 h. On the other hand, strong vasculature destruction after PDT was observed by both Doppler ultrasound (Fig. 9) and immunohistochemistry. Doppler USG examination of irradiated tumors showed a total occlusion of blood flow in large vessels immediately after illumination in both types of PDT studied (Fig. 9).

**Figure 10:**
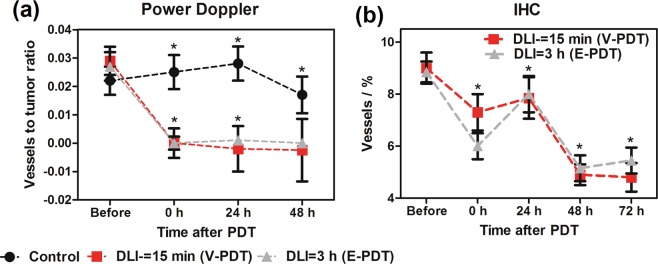
(**a**) Changes in tumor vasculature after PDT as determined from Power Doppler imaging. Values present the ratio of the sum of red pixels (blood flow) to the total tumor area in each image, with SEM. (**b**) Vascular changes in the tumors after PDT as determined from IHC. Average of vessels percentage with SEM is shown for each group. N: 4–12 in each group and given time interval. Results were considered as statistically significant with a confidence level of 95% (p < 0.05).

The amount of large functional vessels decreased from 2–3% to 0%, indicating a total shut-down of these vessels. Evidence indicating the lack of blood flow remained at 24, 48 and 72 h after illumination (Fig. 10). In contrast to Doppler ultrasound, immunohistological detection of endothelial cells shows all vasculature – both functioning and non-functioning, small and large vessels. As shown in Fig. 10b, a gradual decrease in the number of endothelial cells was observed, from 9% in untreated tumors to 5% at 72 h after both DLI = 15 min and DLI = 3 h, indicating destruction of the tumor vascular system.

Large areas of tissue degeneration with many dilated or ruptured vessels were observed in tumor slices at the 48 h time point. At 72 h after treatment, evidence of vascular damage and tissue disintegration was clear, while the level of the vasculature in the control group remained constant at these time points.

**Figure 11:**
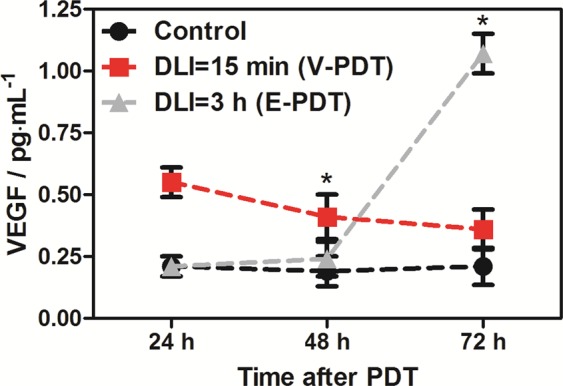
Changes in the VEGF level in tumor lysates 24 h, 48 h and 72 h after illumination as compared to control (K0). Values are averaged from N = 3–6 tumors with SEM. Results were considered as statistically significant with a confidence level of 95% (p < 0.05).

Molecular analysis of VEGF levels (Fig. 11) showed that the vascular damage response molecule is present in treated tissue at a slightly higher level in comparison to control values and shows a dramatic increase at 72 h after DLI = 3 h. In the control group VEGF remained at a constant low level. Most notably, PDT performed at 15 min post PS injection led to progressive decrease in VEGF level which also contributed the final therapeutic outcome. Beginning a few hours after PDT, extensive edema accompanied by areas of noticeable congested tissue were observed, as can be seen both in the photographs and in ultrasound images (Fig. 11).

## Discussion

It is generally recognized that the most popular photosensitizers in clinical practice such as Photofrin and Foscan operate according to Type II mechanism, and in recent years the design of new drugs was focused primarily on improving the production of singlet oxygen. A totally different approach was followed in the development of Tookad Soluble which acts exclusively via Type I mechanism^59^. In this work we applied a series of fluorescent probes to confirm that various types of ROS are generated *in vitro*. The generation of superoxide ions and subsequently hydroxyl radicals contributes to the cytotoxicity of singlet oxygen and their combination gives stronger photodynamic effect leading to the cell death. The cellular uptake is also determinant of PDT efficacy and here again the significant uptake of redaporfin resulted in a greater photodynamic efficacy. Furthermore, redaporfin meets the criteria of the ideal photosensitizer, namely low dark toxicity, selectivity towards tumor tissue shortly after administration, simple formulation and rapid clearance from the body.

Our previous study demonstrated that the pO_2_ tissue level after PDT is an important determinant of tumor response to therapy^31^. Presently, using single-point pO_2_ measurements we have shown that severe and prolonged tumor hypoxia after vascular-targeted PDT leads to a higher percent cure rate. In the present study, our goal was to determine tumor oxygenation after redaporfin-PDT using two approaches, vascular-targeting (DLI = 15 min) and endothelial-targeting (DLI = 3 h). EPR oximetry is a non-invasive method of measuring tissue pO_2_ based on physical interactions between molecular oxygen and molecules of the oximetric spin probe introduced into tissue. The pO_2_ may be determined either spectroscopically or using an imager to map oxygen in studied volume. We have applied a soluble nitroxide given either *i.v*. or intratumorally to acquire a two-dimensional oxygen map of the tumor. Each 2 × 2 mm pixel of the image presents a pO_2_ value, and in the third dimension (depth) the pO_2_ is averaged. We have analyzed both the mean and median value of pO_2_ from all pixels in the image from each tumor and there is a statistically significant difference showing an increase in pO_2_ immediately after both treatments and at 24 h. However, showing a single pO_2_ value is not an appropriate parameter to characterize a map that contains spatial distribution. A more fitting parameter that reflects changes in pO_2_ maps for particular treatment group and time seems to be the hypoxic fraction (HF10), showing a percentage of pixels below 10 torr. However, the best descriptor is the histogram of all of the tumor pO_2_ values that shows a decisive shift in all pO_2_ values in the image. An unexpected increase in pO_2_ post treatment, inconsistent with vasculature shut-down, was observed due to the inadvertent choice of the region of interest in our imaging. Two-dimensional imaging is a preferred choice for small volume tumors, but in the case of extensive edema, it prevents imaging of the tumor only and instead averages over the whole area depth. Additionally, edema can cause changes in temperature, viscosity, pH, or pO_2_ of the tumor microenvironment potentially resulting in an incorrect interpretation of results at the 24 h time point, especially when previously similar experiments using different spin probes show opposite effects. EPR spectroscopy measurement data from 3CP imaging and LiPc probes could vary because of lower sensitivity to oxygen, localized implementation of LiPc crystal versus distribution spin probe in the whole tumor, or averaging pO_2_ value by projecting spectral dimension along tumor. Applying three-dimensional spatial-spectral imaging would be more appropriate in future experiments and would allow for selection of a region of interest and measuring pO_2_ only from tumor tissue.

These results are in agreement with the data published recently for S91 tumors^31^. What is more, at 2 h post PDT, the hypoxic fraction increased significantly and reached the highest values for V-PDT, followed by E-PDT (moderate) and C-PDT. These results reflect the hypoxia within the tumor, not including the edema, and agree with vascular function and density (Figs 9 and 10). Additionally, presented results verified the hypothesis that V-PDT with redaporfin induces strong local hypoxia that may be related with better therapeutic efficacy and tumor growth inhibition. Nevertheless, for cellular-targeted phototherapeutic protocols (E-PDT, C-PDT) the hypoxic area seems to be lower what can lead to intense pO_2_ compensatory effects and modest tumor inhibition^31^. Tong *et al*. also investigated the hypoxia phenomenon after photodynamic treatment (HPPH-PDT) with comparison to static and dynamic PET using hypoxic specific tracer 18F-FMISO combined with pharmacokinetics modeling in U87MG and MDA-MB-435 tumors. These data clearly indicated difference in tumor hypoxia in response to PDT and suggest that monitoring intrinsic and PDT-induced hypoxia can be considered as prognostic factor for further therapeutic efficacy evaluation^29^.

Doppler ultrasound blood flow analysis showed no differences between the two applied V-PDT and E-PDT protocols. In both groups of tumors, we observed a total arrest of blood flow in large vessels immediately after treatment. Together with accompanying edema and erythema of the illuminated area, this indicates an immediate effect of therapy with strong local response^60^. A total lack of blood flow was seen for next 48 h. Therefore, similar to other common photosensitizers, such as Photofrin, application of vascular-targeted and endothelial-targeted PDT with redaporfin brings about instant and prolonged inhibition of tumor perfusion^61^. Immunohistological staining on endothelial cells confirmed destruction of tumor vasculature after therapy. This decrease was gradual and more pronounced at 48 and 72 h. Vascular tissue breakdown was associated with degradation and necrosis of tumor tissue. Well-known effects of PDT, especially vascular-targeted PDT, are hypoxia and oxidative stress. Both of these factors contribute to increased VEGF expression in tumor tissue^22,62^. We noticed a substantial increase in VEGF at 72 h after DLI = 3 h but not after DLI = 15 min. This suggests that despite very similar effects on vasculature, the two protocols exert different effects at the molecular level. One explanation might be that at DLI = 3 h the PS has longer to diffuse into cells and damage is more extensive, causing a higher response in VEGF stimulation. Another possibility is that DLI = 15 min caused such extensive tumor cell death, the cells were unable to respond by protein production. It is important to note that higher VEGF levels can lead to stimulation of angiogenesis and tumor regrowth.

PDT protocols examined led to tumor vasculature shut-down and endothelial cells destruction, likely leading to tumor hypoxia as evidenced by VEGF production, especially after DLI = 3 h. The observed paradoxical increase in pO_2_ levels after PDT resulted from an imaging approach which included well-oxygenated areas of tumor edema that developed immediately after treatment. A significant difference in survival was observed between animals treated with a DLI = 3 h, DLI = 72 h protocol (about 25% of animals without tumor regrowth) and a 15 min DLI protocol (67% cure rate). This result indicates a significant advantage of V-PDT. Its outcome also corresponds to a high amount of PS in blood and within tumor blood vessels shortly after administration (15 min). Our studies also confirmed that a key factor for efficient PDT is a complete closure of tumor vessels. In order to achieve this outcome, short DLI protocols should be applied. Moreover, due to tumor hypoxia, PS acting via Type I photochemical mechanisms, which are less independent on O_2_ level, should be used in V-PDT.

## Methods

### Photosensitizer

Redaporfin (synthetic bacteriochlorin derivative, also known as F_2_BMet or LUZ11) was kindly provided by Luzitin S.A, Coimbra, Portugal, as a powder samples transported under appropriate atmosphere conditions. Redaporfin-CrEL formulation was prepared by dilution of a concentrated solution of CrEL:EtOH (1:5) in PBS to obtain CrEL:EtOH:PBS (0.2:1:98.8 v/v/v)^4,46,63^. As an administration vehicle the Cremophor EL micelles in saline solutions was used. The final dilution of Cremophor EL/ethanol mixture at applied non-toxic dose was subsequently injected into mice intravenously via tail vein at dose of 1.5 mg/kg B.W. The injected volume varied between 0.2 ml and 0.5 ml depending on individual’s animal weight, either 15 minutes or 3 hours prior to illumination. The absorption spectra of redaporfin were measured using HP8453 spectrophotometer in 1 cm path-length quartz cell. Fluorescence measurements were performed using Perkin Elmer Fluorescence Spectrometer LS 55 with λ_ex_ = 505 nm and λ_em_ = 700–800, respectively.

### *In vitro* evaluation

*In vitro* evaluation was performed using the experimental conditions and overall procedures described in our previous work^48^.

#### Cell culture

Lewis lung carcinoma (LLC) cells were grown in RPMI-1640 with addition of 10% FBS and supplemented by antibiotics (100 IU/mL penicillin and 100 mg/mL streptomycin). The cells were cultured in incubator maintained at 37 °C with 5% CO_2_ under fully humidified conditions. All experiments were performed on cells in the logarithmic phase of growth. Media were replaced every 2 days and cells were subcultured using 0.25% trypsin-EDTA^48^.

#### Cellular uptake

To estimate the possibility of efficient accumulation of photosensitizer in LLC cells the time-dependent cellular uptake experiment were performed. LLC cells were seeded on 96-plate microplate (10^4^ per well). After 24 h, the cells were incubated with redaporfin (5 μM) for time intervals from 2 h up to 24 h. Redaporfin solutions were prepared by diluting its stock solution in DMSO with the culture medium to the desired final concentration (5 μM). The content of DMSO did not exceed 0.5%. After each incubation time, the cells were washed twice with PBS and solubilized in 30 μL of Triton X-100 and 70 μL of DMSO/ethanol solution (1:3). The accumulation of redaporfin was detected by fluorescence measurements (λ_exc_ = 505, λ_em_ = 750 nm) with the microplate reader (Tecan Infinite M200 Reader)^48^.

#### Dark cytotoxicity and cells viability assay

To assess the dark cytotoxicity of redaporfin, LLC cells seeded on 96-plate microplate (10^4^ per well) were incubated with redaporfin solution prepared in growth medium in concentrations from 0 to 100 μM. Treated cultures were incubated for ca. 24 h in the dark. Next, the redaporfin solution of each well was removed, cells were washed in PBS and fresh culture medium supplemented with FBS and antibiotics was added to each well and cells were returned to the incubator for 24 h. Next, the cells viability was calculated based on the MTT and AlamarBlue assay. The MTT (3-(4,5-dimethylthiazol-2-yl)2,5-diphenyl tetrazolium bromide) and AlamarBlue assays were used to quantify cell survival after photodynamic effect. MTT or AlamarBlue dissolved in PBS at content 10% of final solution were added to each well and the microplates were further incubated for ca. 3 h. In the case of MTT, medium was then discarded and 100 μL of mixture of DMSO/methanol (1:1) were added to the cultures and mixed thoroughly to dissolve the dark blue crystals of formazan. Formazan quantification was performed using an automatic microplate reader (Tecan Infinite M200 Reader) by absorbance measurements with a 565 nm test wavelength. Resorufin quantification was performed using by fluorescence measurements with a 605 nm test wavelength^48^.

#### Photodynamic effect

The nontoxic concentration of redaporfin (15 μM) was used for cells incubation. Cells were incubated for 3 h in the dark with redaporfin solution or with in a culture medium (RPMI1640 supplemented with 10% FBS and antibiotics). After this incubation time, the cells were washed two times with PBS with Ca^2+^ and Mg^2+^ and irradiated with a 735 ± 20 nm LED light for various time intervals. Next, the cells were washed with fresh medium and the plates were returned to the incubator for 24 h. Cell viability was determined by MTT and AlamarBlue assays in independent experiments performed 24 h post-irradiation. Images from the cell morphology before and 24 h post-PDT were obtained using optical microscope (Olympus)^48^.

#### Flow cytometry analysis

Cell death after photodynamic effect was quantified for selected condition using the AnnexinV-FITC/PI double staining kit (Sigma Aldrich). The cell death of LLC cells was investigated 4 h after photodynamic treatment. Briefly, the LLC cells (1 · 10^5^ per well) were cultured overnight in 12-well plates. Then cells were subjected to redaporfin-mediated photodynamic effect (described above), collected by centrifugation and washed twice in Hank’s Balanced Salt Solution (HBBS). The cells were then resuspended in 500 μL binding buffer and stained with annexin V-FITC and PI according to the manufacturer’s instructions (Sigma Aldrich). Stained LLC cells were then examined using Guava® easyCyte™ flow cytometer. Obtained data were analyzed using FlowJo 10.5.3 software (BD Bioscience).

#### Detection of reactive oxygen species

The 3′-p-(aminophenyl)fluorescein (APF), 3′-p-(hydroxyphenyl)fluorescein (HPF), Singlet Oxygen Sensor Green (SOSG) and dihydroethidium (DHE) fluorescent probes were employed for detection of reactive oxygen species formation during illumination. LLC cells were incubated with 15 μM redaporfin solution prepared in OptiMem for 3 h. Two hours prior to the end of incubation each probe at concentration at 20 μM was added to the solution In case of SOSG, cells were incubated with standard maintenance medium (SMM)-based solutions^64,65^. Then, cells were washed with PBS with Ca^2+^ and Mg^2+^ and irradiated with the 735 ± 20 nm light source for various time intervals. The probes fluorescence signals (for APF and HPF: λ_exc_ = 488 nm, λ_em_ = 515 nm, for SOSG: λ_exc_ = 505 nm, λ_em_ = 525 nm and λ_exc_ = 480 nm, λ_em_ = 580 nm for DHE, respectively) were determined using a microplate reader (Tecan Infinite M200 Reader) immediately before and after illumination^48^.

### Animal and tumor model

Male C57BL/6J mice, initially 8–12 weeks old and weighing approximately 20 g were originally obtained from the animal breeding facility at Faculty of Biochemistry, Biophysics and Biotechnology of the Jagiellonian University (Cracow, Poland). All experimental procedures were approved by First Ethic Local Committee of Jagiellonian University (permission no 2/2015 and 190/2018) and experiments were performed in accordance with the relevant guidelines and regulations. Mice were housed in standard laboratory conditions with LD:12/12, humidity: 60%, temperature: 23 °C with unlimited access to standard chow diet and drinking water. The Lewis lung carcinoma (LLC) cells were growing as a monolayer at 37 °C in a humidified atmosphere of 5% CO_2_/95% air in RPMI-1640 supplemented with 10% heat-inactivated fetal bovine serum (BioTech, Poland) and penicillin-streptomycin under sterile tissue culture conditions. The final suspension of tumor cells ready for intradermal injection was estimated at 0.5 million per 1 µl of PBS solution and in that form introduced into the skin of the right hind leg. Tumor selection to experiments was performed when tumor implants reached the size of 30–50 mm^3^ after 7–9 days, and then the animals were randomly assigned to one of the following groups due to the protocol applied afterwards: DLI = 15 min, DLI = 3 h, DLI = 72 h and K0- control group. The therapeutic procedures were started when tumors reach more than 0.5 cm in each diameter (which corresponds to tumor volume about 80–100 mm^3^). LLC is a well described mouse solid tumor, growing in an encysted form, therefore it is known to cause no significant discomfort to the tumor bearing animals. The growth of the tumor was monitored by caliper every 24 hours and the volume was calculated.

### Biodistribution

LLC cells were implanted subcutaneously into the right thigh of male C57BL/6J mice (as described above). The *i.v*. injection of photosensitizer was done in each animal at a dose of 1.5 mg·kg^−1^ into the tail vein of each animal and tissue distribution was evaluated at 15 min, 3 h and 72 h post-administration. In general, experiments were performed according to procedure described in our previous work^48^. At appropriate time post-injection, the mice were anesthetized with ketamine and xylazine and sacrificed. For each animal, selected organs and tissue samples were collected separately and weighed. The content of redaporfin in the tissue samples was determined by fluorescence measurements. To extract the PS, tissue samples were homogenized in EtOH/DMSO solution (75:25) using a Yellowline by IKA DI 25 basic animal tissue homogenizer. The homogenates were centrifuged, the supernatants were collected, and the pellets was re-extracted four more times using the procedure described above to ensure complete recovery of the drug. The extracts were pooled and the fluorescence analysis of the extracts was done. The samples were excited at 505 nm, and the fluorescence spectra were recorded in a range of 600–800 nm. The amount of redaporfin in the tissues was reported as the average from four animals (with the standard error of the mean, SEM). The redaporfin concentration was estimated based on fluorescence intensity of redaporfin in prepared samples and comparison with the calibration curve^48^.

### Photodynamic therapy *in vivo*

Prior to therapeutic procedure the tumor leg was shaved carefully with a razor blade in the area of the tumor and surrounding tissue. The photodynamic therapy was performed according to procedure described in our previous work^48^. The tumor illumination was performed at DLI = 15 min, DLI = 3 h and DLI = 72 h using a NIR-laser light (Omicron laser model LDM750.300.CWA.L.M equipped with optical fiber model FD/Medlight (Ecublens, Switzerland) was used as a light source) at 750 nm, light dose at 105 J·cm^−2^ and a laser power of 130 mW. The illuminated area with a diameter of 1.3 cm was kept constant during irradiation. Survival curves were determined by Kaplan− Meier analysis that present the survival data for a certain amount of time after photodynamic treatment.

### *In vivo* Power Doppler imaging

High Resolution Ultrasound Imaging System, devoted to small experimental animals (Visual Sonics Vevo 2100) with MS-550D serial transducer was used. During imaging body temperature was controlled by heating, and maintained at 37 °C. Anesthesia was induced by 3 vol% isoflurane (Aerrane, Baxter Polska Sp. z o. o., Poland) and then maintained at 1.5–2.0 vol% isoflurane in the air, delivered at 1.2 l/min. For the confirmation of the tumor position, a B-mode ultrasound (grey scale) was performed with a central frequency 40 MHz. Power Doppler (PD) ultrasound contain color imaging of vascularization was performed with central frequency 32 MHz and pulse repetition frequency (PRF) 3–4 kHz. Two-dimensional tumor sections with a field of view (FOV) 12 × 12 mm were collected along the third tumor dimension using motorized transducer holder, resulting in a 3D image. The in plane (XY) resolution was up to 15 μm and Z resolution was equal 200 μm. The volume of the vasculature was calculated as a sum of pixels of functional vessels detected in each image slice. The tumor volume was a sum of pixels of tumor area marked by hand on each image slice.

### EPR imaging

Approximately 10 minutes prior to measurements, animals were sedated and anesthetized with inhaled anesthesia as describe in Power Doppler Imaging section. Animal temperature was maintained at 37 °C by using a thermal blanket. 3D EPR spatial spectral imaging was conducted using Elexys 540 L-band continuous wave system (Bruker, Germany) with a surface coil. Two spatial and one spectral dimension imaging allowed obtaining 2D images of spin probe distribution, and for each pixel whole EPR spectrum was acquired. The tumor size was approximately 4–6 mm in thickness, and at L-band the surface coil acquires information from up to 6 mm. Therefore, the collected 2D data was a sum of oximetric spin probe signal in the volume of around 1 cm^3^ in the direct proximity of the coil. As a spin probe 3-carboxyl proxyl (Sigma Aldrich) was used. The spin probe was injected at volume of 200 μl (9 mg/ml) intraperitoneally before and right after PDT or directly into the tumor at volume of 10 µl at 24, 48 and 72 hours after therapy. EPR measurement parameters were as follows: amplitude modulation 0.7G, modulation frequency 30 kHz, sweep time was around 1.5 s, 10.75 mW microwave power. For imaging low field line with center field 390.95G was used. Spatial – spectral imaging was performed with two spatial and one spectral dimension. Maximum gradient was set as 3G/cm. Total number of projections was 434 (31 spatial and 14 spectral). Total imaging time was around 30 minutes.

### Image reconstruction and post-processing

Images were reconstructed using FBP from EPR-IT software with homebuilt modifications. 512 points per spectrum were acquired and then subsampled to 128. The number of projections were interpolated by factor of 4. Projections were filtered using Ram-Lak filter with cutoff equal 0.5. Spatial field of view FOV = 4.24 cm and spectral field of view was 5G. Image size was set to 128 pixels in each dimension. For spectrum from each pixel peak to peak linewidth distance was calculated and converted to pO_2_ value. Calibration curve was performed for three pO_2_ values: 0, 3 and 21% of oxygen in 3CP solution. Finally, spatial images of spin probe distribution were converted to 2D oxygen maps using calibration curve. Spatial resolution was less than 2 mm as determined earlier using phantom imaging. Hypoxic fraction 10 (HF10) was calculated as the number of pixels value less or equal to 10 torr divided by number of all pixels in our region of interest.

### Immunohistochemistry with pimonidazole and confocal imaging

Immunohistochemical staining was performed to observe *ex-vivo* tissue hypoxia with Hydroxyprobe^TM^ kit (Hydroxyprobe Inc., Burlington, MA). Multiple mice bearing LLC tumors (N = 5) bearing LLC tumors were intravenously injected via tail vein with 60 mg/kg BW of pimonidazole before and 2 h after PDT treatment (described above)^29^. After 90 min of incubation of pimonidazole, mice were euthanized with ketamine/xylazine and tumors were harvested. Tumors were weighed, snap-frozen, and stored at −80 °C. Harvested tumors were then taken in a cryostat (Leica) and cut into 5 μm-thick sections. Sections are placed on microscope slides for staining based on the manufacturer’s instructions. Frozen slides were air dried (30 min) and fixed with ice-cold acetone for 10 min. After acetone evaporation (30 min) slides were washed two times with PBS and then incubated for 10 min in 0.3% H_2_O_2_ in MeOH/PBS. Then, slides were washed two times (5 min) with PBS and blocked 1 h in blocking buffer (1× TBS, 0.3% Triton X-100, 5% FBS). The blocking solution was removed and slides were incubated with add FITC-conjugated anti-pimonidazole primary antibody overnight at 4 °C. After incubation, slides were washed three times (5 min) with TBS-0.01% Tween 20, stained with Hoechst33342 for 10 min, rinsed with PBS, mounted with prolong gold mounting solution and coverslip. Prepared tissues were imaged with fluorescence confocal microscopy LSM880 (Carl Zeiss) with 40× magnification as desired and analyzed using ZEN software (Carl Zeiss). For further analysis, five complete and non-overlapping regions of interest (ROI) were randomly selected from each prepared slide. All microscopic images were adjusted with the same parameters; thus, tumor hypoxia was reflected directly by the pimonidazole staining color intensity (green signal).

### Protein analysis and immunohistochemistry staining (IHC)

Enzyme immunoassay - Quantikine M mouse ELISA kit was used to quantify vascular endothelial growth factor (VEGF) levels in control and treated tumor lysates (R&D Systems, Minneapolis, MN). Tumors were homogenized in lysis buffer and sonicated. Buffer lysates obtained from whole dissected tumor tissue were analyzed for total protein level and subsequently processed following the manufacturer’s protocol. The resulting amount of protein was presented as pg of VEGF per 1 mg of total protein content. Tumors previously fixed in cryoprotectant were cut into 5 µm slices for immunohistochemistry. 9F1 Rat monoclonal antibody against mouse endothelium (Radbound University Nijmegen Medical Centre) was used to visualize vessels. The level of secondary antibody presentation was assessed by counting the number of dark pixels in relation to the total pixel number of the whole image in 3–5 hot spots in each slice from each tumor 2–3 representative slices were microscope images were analyzed.

### Statistics

For EPR imaging and oximetry statistical data analysis two-way ANOVA was performed. As a factors treatment time and treatment group were used. RiR-Tukey post-hoc test for difference number of elements in particular group did not show statistical differences for p = 0.05. For other experiments, the t-test was applied for the evaluation of statistical significance (p values). Results were considered as statistically significant with a confidence level of 95% (p < 0.05). Statistical analysis was performed with the STATISTICA12.5 software (StatSoft Poland, Kraków).
